# Initial In Vivo Evaluation of a Novel Left Ventricular Assist Device

**DOI:** 10.1155/2015/148579

**Published:** 2015-10-11

**Authors:** Guanghui Wu, Changyan Lin, Haiyang Li, Xiaotong Hou, Chen Chen, Xiujian Liu, Chuangye Xu, Jing Wang, Peng Yang, Wenbo Qu

**Affiliations:** ^1^Beijing Institute of Heart Lung and Blood Vessel Diseases, Beijing Anzhen Hospital, Capital Medical University, Beijing 100029, China; ^2^Beijing Anzhen Hospital, Capital Medical University, Beijing 100029, China; ^3^CH Biomedical Incorporation, Jiangsu 215125, China

## Abstract

The aim of the study was to use the ovine model to evaluate the hemocompatibility and end-organ effects of a newly developed magnetic suspension centrifugal left ventricular assist device (LVAD) by CH Biomedical Inc., Jiangsu, China. The LVADs were implanted in 6 healthy sheep, where inflow was inserted into the left ventricular apex and outflow was anastomosed to the descending aorta. All sheep received anticoagulation and antiaggregation therapy during the study. Hematologic and biochemical tests were performed to evaluate anemia, hepatorenal function, and the extent of hemolysis. The experiments lasted for up to 30 days on the beating hearts. All sheep were humanely killed at the termination of the experiments, and the end-organs were examined macroscopically and histopathologically. Autopsy was performed in all animals and there was no thrombus formation observed inside the pump. The pump's inflow and outflow conduits were also free of thrombus. Hematologic and biochemical test results were within normal limits during the study period. Postmortem examination of the explanted organs revealed no evidence of ischemia or infarction. Based on the in vivo study, this LVAD is suitable for implantation and can provide efficient support with good biocompatibility. The encouraging results in this study suggest that it is feasible to evaluate the device's long-term durability and stability.

## 1. Introduction

The heart failure epidemic is a largely unabated problem, with more than 26 million people affected worldwide [1]. Although optimal drug therapy has been shown to benefit patients with heart failure, the prognosis and quality of life of the patients remain extremely poor. Heart transplantation is the only curative treatment for end-stage heart failure; however, it is only available to few patients because of the shortage of donor hearts [2]. Many types of implantable LVADs have been developed since the early 1960s, which have been widely used in heart failure patients as a bridge to recovery, transplantation, or destination therapy [2–5]. Compared with other types of pumps, the third-generation implantable LVADs suspend the impeller with magnetic or hydrodynamic suspension systems [6–9]. Because of simpler design (no mechanical bearings, no mechanical or biological valves), these devices show a potential of longer durability. In addition, the reduced dimensions and integrated inflow cannula allow these devices to be completely implanted into the pericardial cavity and be directly adjacent to the heart, thereby avoiding the abdominal surgery that is generally required to implant competing devices. Moreover, the reduced procedural invasiveness is expected to lead to more rapid postoperative recovery.

Few LVADs are clinically used in China partly because of high prices. Development of a less expensive pump for clinic use in China is urgently needed. We designed and manufactured a magnetic suspension third-generation implantable LVAD and conducted experiments to evaluate the hemocompatibility and end-organ effects of the device.

## 2. Materials and Methods

### 2.1. Animal Care

Six healthy adult sheep (weighing 50.1 ± 5.8 kg) were used in this study. All animals were purchased from Beijing Pinggu Animal Experimental Center (permit number: SYXK (Beijing) 2010-0019) and received humane care in compliance with the “Regulation to the Care and Use of Experimental Animals” (revised 2006) as published by the Beijing Council on Animal Care. The study protocol was approved by our Institutional Animal Care and Use Committee.

### 2.2. Device Description

**Figure 1:**
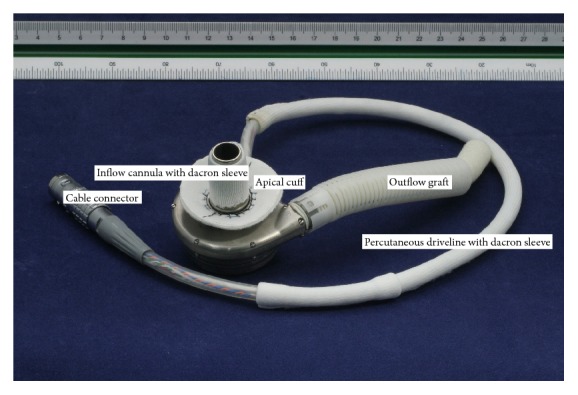
The LVAD.

**Figure 2:**
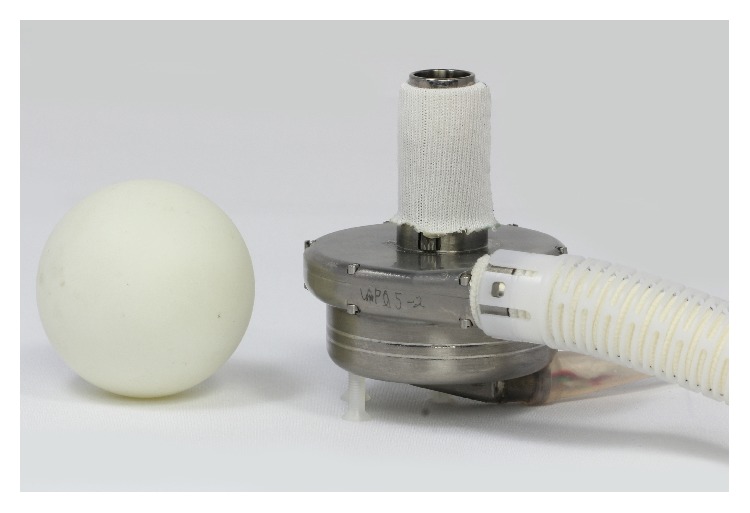
Comparison between of the LVAD and a table tennis ball.

**Figure 3:**
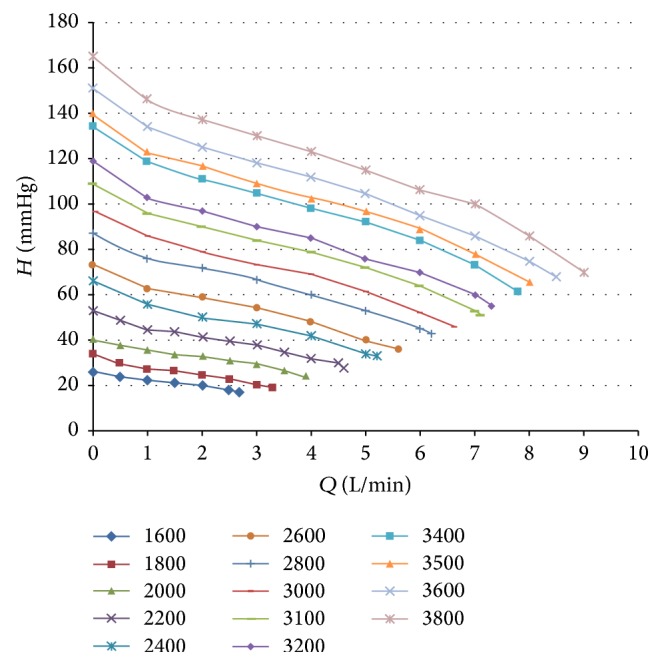
Pressure-volumetric flow rate curve of the LVAD.

The LVAD is a small, wearless, ultracompact centrifugal blood pump (Figure 1). Computational fluid dynamics (CFD) analysis was used in the design of the pump. Overall size of the prototype device is 42 mm in diameter and 22 mm in height.  Figure 2 shows the comparison between the LVAD and a table tennis ball. The entire device has a displaced volume of 45 cc, weighs about 130 grams, and can deliver flow up to 10 L/min (Figure 3). The cable connector of the percutaneous driveline connects to an external controller, which sends power and operating signals to the blood pump and collects information from the pump. The apical cuff, comprised of a titanium frame surrounded by a polyester-covered felt ring, is used to secure the inflow cannula to the LV apex. The LVAD has a short integrated inflow cannula which is inserted into the left ventricle and the pump is positioned in the pericardial space. The 10-mm outflow graft is anastomosed to the descending aorta. External system components include a microprocessor-based controller, a monitor, lithium-ion battery packs, alternating current and direct current power adapters, and a battery charger.

### 2.3. Anesthesia

We followed a standard institutional anesthesia protocol that has previously been described in detail [10]. Before the surgery, sierra triazine hydrochloride (0.5 mg/kg) was intramuscularly injected into each animal. Intravenous injection of ketamine (15 mg/kg) was then administered to induce anesthesia. A venous catheter was inserted into the left ear vein to establish an intravenous access. Each animal was mechanically ventilated after endotracheal intubation. General anesthesia was maintained with isoflurane (3%) in oxygen (100%) and continuous infusion of propofol (6~8 mg/kg/h).

### 2.4. Surgical Technique

**Figure 4:**
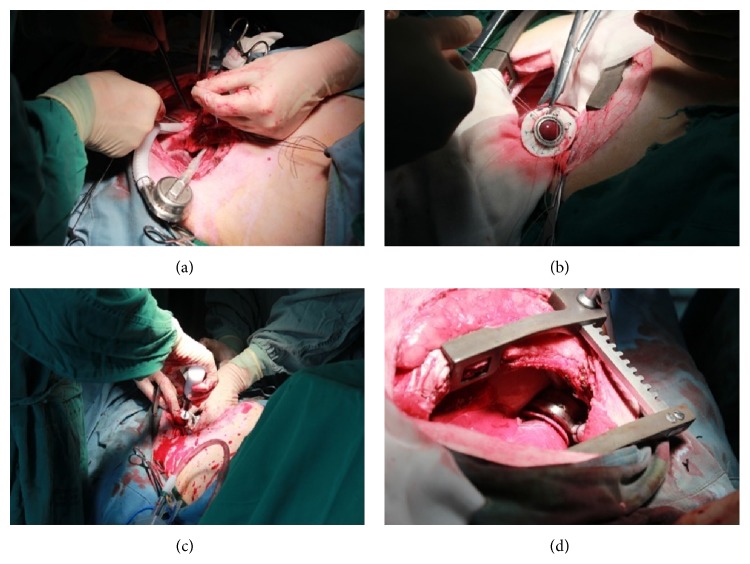
The process of the pump implantation: (a) outflow graft anastomosis; (b) suturing the sewing ring; (c) removing the LV core; and (d) implanted position of the pump.

Each sheep was placed in the right lateral decubitus position for a left thoracotomy, while its electrocardiography was monitored. An arterial pressure line was then introduced in the left carotid artery for intraoperative and postoperative blood pressure monitoring. A lidocaine drip (2 mg/min) was initiated to prevent arrhythmias, and intravenous injection of vecuronium bromide (5 mg) was administered to maintain muscle relaxation. A left subcostal thoracotomy was performed at the 4th intercostal space. The pump was filled with saline and restarted to work at least for 5 minutes in order to ensure normal working conditions. The driveline was tunneled to exit near left paraspinal area at the 8th intercostal space and was connected to the external controller. The pericardium was incised after fully exposing the left ventricular apex. The descending thoracic aorta was dissected for outflow graft anastomosis. Heparin (1.5 mg/kg) was given intravenously to keep whole blood activated clotting time (ACT) above 480 s. Sodium nitroprusside (0.5 mg/kg/min) was injected intravenously to control the blood pressure. The descending thoracic aorta was clamped with a side biting clamp (Geister, Inc., Germany) and the 10 mm artificial blood vessels graft (Vascutek-Terumo), which was cut to proper length, was anastomosed with continuous 4-0 Prolene (Ethicon, Inc. USA) suture. The apex was elevated and a sewing ring was sutured circumferentially, to the apex with 10 to 12 interrupted 2-0 Ethibond pledgeted (Johnson, Inc., USA) sutures. Using a scalpel, a cruciform incision was made in the apex on the beating heart. Then the LV core was removed by a conical coring knife through the ventricular sewing ring, and the inflow cannula was inserted into the left ventricular cavity quickly and secured. The LVAD was activated and the pump was deaired through a small-diameter needle inserted into the outflow graft with a pump speed of about 1900 rpm; then the motor speed was initially adjusted to achieve maximum heart unloading.  Figure 4 shows the process of pump implantation of case 4.

During the surgery, penicillin (4.8 million units) was injected intravenously to prevent infection. Protamine was then administered to reverse the effects of heparin. One chest tube was inserted into the pleural cavity, which was closed in accordance with standard technique.

### 2.5. Postoperative Care

After extubation, the sheep was transferred into a specially designed cage in the observing room for continuous monitoring of heart rate, aortic pressure, and pump flow. Heparin was continuously infused to maintain the activated clotting time (ACT) within 150~180 s, and afterward oral administration of warfarin was used to maintain international normalized ratio (INR) of 1.2~2.0. Injection of penicillin (3.2 million units) was performed daily to prevent infection until the white blood cell count was within normal range. Two mg/kg/h lidocaine was continuously infused in the first two postoperative days (PODs) through the subcutaneous wound catheter for analgesia. Observation of appetite, urine volume, body temperature, respiratory rate, and neurological status was performed daily for evaluating the general status of the sheep.

### 2.6. Data Collection

During the study, blood samples were taken routinely from the carotid artery for hematologic and biochemical tests. These tests were repeated daily for 10 days and then every 5 days until the end of the study. The pump operating parameters, such as pump flow, pump speed, and power consumption, were recorded continuously by the control software. In addition, the body surface temperature was also measured daily throughout the study.

### 2.7. Macroscopic Analyses

Heparin (3 mg/kg) was administered just before the sheep were humanely killed in order to prevent postmortem clot formation in the pump. In each case, the LVAD was explanted, opened, and photographed. Any infarcts or focal lesions in any tissue were noted during gross evaluation. The pump housing and impeller were carefully inspected for fibrin formation or thrombus. The inflow and outflow grafts, the aorta, and the ventricle at each cannulation site were also evaluated and photographed.

### 2.8. Histopathology Evaluation

Routine histologic examination was performed on the heart, lungs, liver, kidneys, and spleen. Blocks of tissue were immersion-fixed in 10% neutral-buffered formalin with a tissue-to-volume fixative ratio of 1 : 10. After 72 hours of fixation, an illustration of the sectioning site was made for each device. Cross sections of the soft-tissue interface with the device were processed with standard paraffin. Two 5-micron-thick sections from each of the sampled regions were stained with hematoxylin-eosin stain.

## 3. Results

### 3.1. Animals

All six sheep successfully underwent LVAD implantation in 3 hours without CPB and recovered from anesthesia without complications within the first 2 hours. All physiological indexes, including heart rate, respiratory rate, and body temperature, remained within normal ranges during the study period. None of the sheep developed anorexia, respiratory disorders, or neurological disorders.

### 3.2. Device

No notable device-related problems occurred during the whole experiments. Average pump data values ± SD included an average speed of 3400 ± 50 rpm, average flows (simulation) of 5.03 ± 0.6 L/min, and average power consumption of 4.5 ± 0.5 watts.

### 3.3. Clinical Chemistry and Hematologic Data

Values at baseline and at postoperative days 1, 5, 10, 20, and 30 were shown in Table 1. All sheep had an increased WBC count during the immediate postoperative period; but the WBC returned to normal ranges at day 30. Expected decreases in red blood cells and hematocrit were noted in all sheep postoperatively, and the hematocrit level did not return to the normal range at the termination of the experiments. The plasma free hemoglobin (FHB) levels increased to the peak value at the fifth day after the surgery and then gradually reduced to the preoperative level. Clinical biochemistry values indicated normal liver and renal function.

### 3.4. Macroscopic Analyses

**Figure 5:**
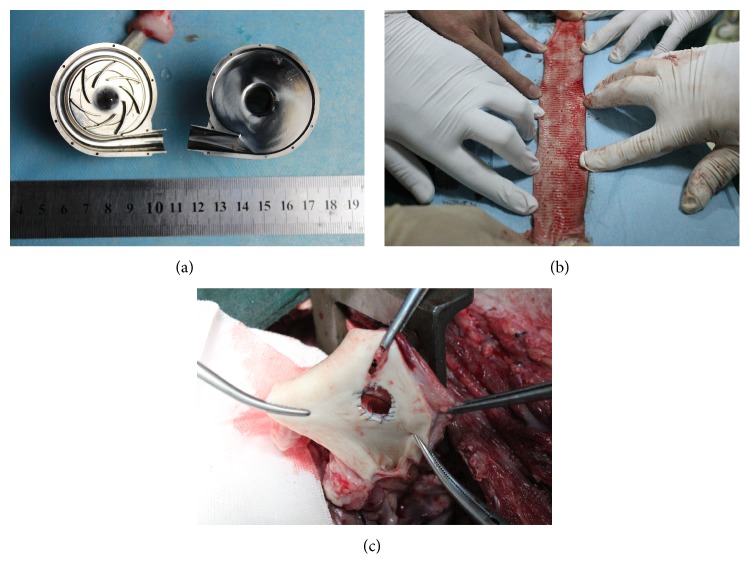
Pump, blood flow channels: (a) pump body; (b) the outflow graft; and (c) the arterial anastomotic stoma.

Gross examination of the pump inflow conduits, the pump interiors, and the impeller surfaces from all sheep showed no remarkable thromboembolic fragments. Necropsy of the main organs of all sheep revealed no lesions.  Figure 5 shows the gross examination of the pump, the outflow graft, and the arterial anastomotic stoma of case 5.

### 3.5. Histopathology Evaluation

**Figure 6:**
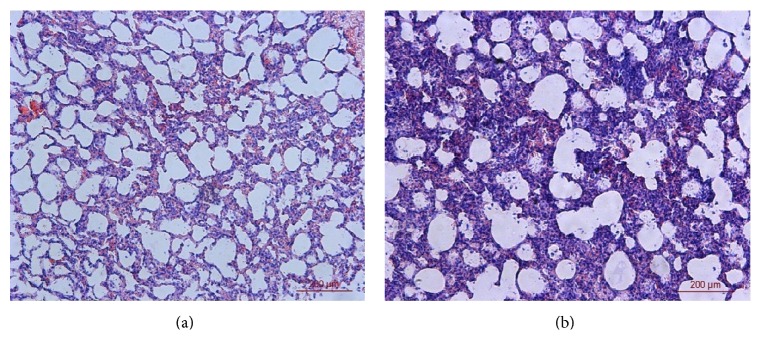
The histopathologic results of the diseased lungs (HE, 200x).

**Figure 7:**
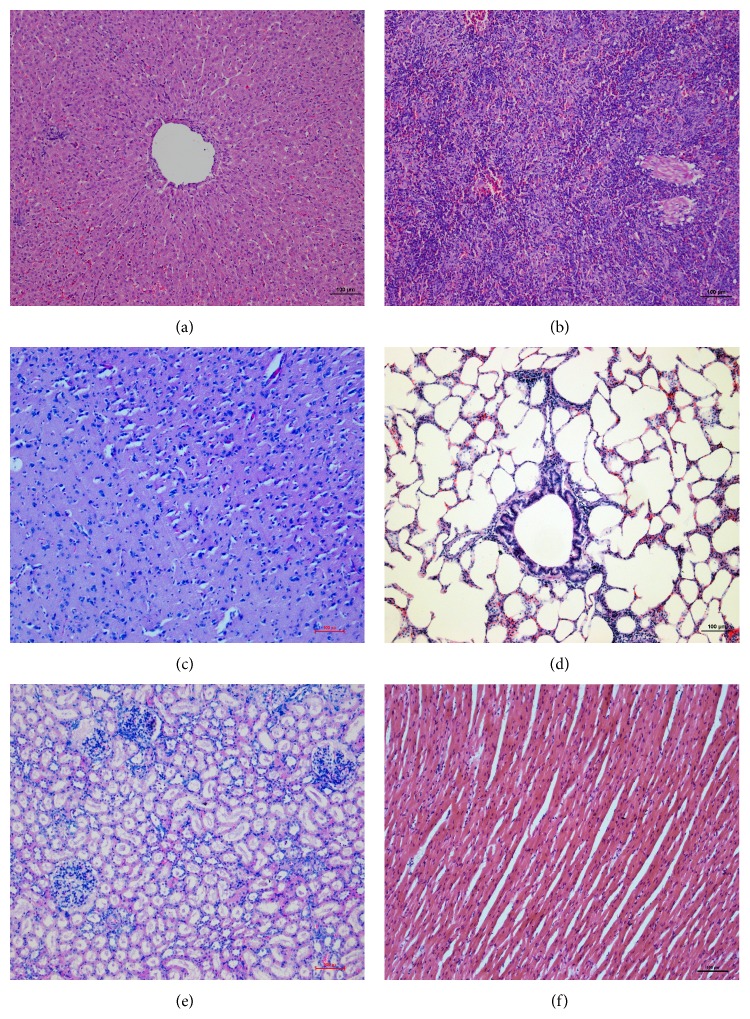
The histopathologic results of case 5 (HE, 100x): (a) liver; (b) spleen; (c) brain; (d) lung; (e) kidney; and (f) myocardium.

The left lung close to the outflow graft protective sleeve parts had lobular pneumonia in all sheep. Histologic examination (Figure 6) showed pulmonary cell edema, breaks in the alveolar walls, marked inflammatory cell infiltration, and loss of some capillaries in the lesion partly because of the minor atelectasis of the left lung lobe adjacent to the outflow cannula. Histopathology results of the remaining organs showed no obvious lesions.  Figure 7 shows the histopathologic results of case 5.

## 4. Discussion

The LVAD demonstrated appropriate blood-handling characteristics and reliability in the ovine model for 30 days. The extent of hemolysis was demonstrated to be qualitatively negative throughout the experiments. There was no observation of anemia or hyperbilirubinemia. Therefore, the extent of hemolysis was considered to be within a tolerable range. In contrast to the HeartWare VAD (HeartWare Inc., USA), which utilizes the passive magnetic and hydrodynamic thrust bearings as the hybrid suspension system to drive the impeller, our blood pump utilizes a fully electromagnetic suspended system to create a contact-free rotation of impeller. Moreover, the device here is lighter than the HeartWare VAD. Miniaturization may enable less invasive surgical techniques, treatment of patients with nonstandard cardiac anatomy, right and left ventricular support, and support for a less ill cohort of patients [11]. The magnetic levitation systems also maximize the antithrombogenicity and the durability of the blood pump. When higher pump flows are required, conventional axial flow pumps have higher rotational speed in comparison with centrifugal pumps because of lower torque generation [12]. Therefore, conventional axial flow pumps generally have a mechanical wear problem associated with the rotational speed. Mechanical wear limits the durability, which makes the prognosis in patients with LVAD [13, 14] difficult.

Hemorrhagic complication is one of the major events observed during LVAD support [15, 16]. It has been reported that the rate of hemorrhagic events associated with LVAD is almost ten times the rate of thromboembolic events [17]. Excessive anticoagulation and antiaggregation therapy initiates hemorrhagic complications. In this study, based on early in vitro hemolysis test results (NIH 0.00067 ± 0.00037 mg/dL), we selected lower anticoagulation strategies, and the target ACT range of this study remained at (157 ± 28) s. Since this study was terminated with no evidence of hemorrhagic complications or thrombus formation inside the pump, the current anticoagulation and antiaggregation therapy is considered to be at an appropriate degree.

Most LVADs require partial or total cardiopulmonary bypass support for LV apex cannulation, which increases the surgical time and risk and can further compromise ventricular function in patients who have acute heart failure [18]. The pumps developed in this study were implanted while the hearts were beating. The technique here reduced the surgical time to within 1 hour and prevented the deleterious effects of cardiopulmonary bypass.

The Thoratec HeartMate II (Thoratec Inc., USA) and the HeartWare HVAD (HeartWare Inc., USA) are currently the 2 most commonly implanted LVADs worldwide [19]. Animal tests of the HeartMate II suggest that its operational goal of 5 years can be met [20]. The HeartWare HVAD system also demonstrated appropriate blood-handling characteristics and reliability in the ovine model for 90 days [21]. In future studies, long-time animal experiments will be conducted to evaluate the hemocompatibility, biocompatibility, performance, and safety of our pump, the results of which will be compared to the literature.

## Figures and Tables

**Table 1:** Mean preoperative and postoperative hematologic and biochemical data.

	Preoperative	Day 1	Day 5	Day 10	Day 20	Day 30
Hematologic data (mean ± SD)						
White blood cells ×10^9^/L	10.3 ± 1.6	29.9 ± 11.3	22.3 ± 8.7	15.6 ± 4.6	10.3 ± 1.6	9.6 ± 1.3
Red blood cells ×10^12^/L	7.9 ± 1.6	5.7 ± 1.1	6.2 ± 0.8	7.1 ± 0.7	7.7 ± 1.1	8.1 ± 0.3
Hemoglobin, g/L	107 ± 12.6	96 ± 7.7	91 ± 6.8	68 ± 5.0	91 ± 9.3	97 ± 8.2
Hematocrit, %	31.14 ± 1.05	25.56 ± 2.03	25.34 ± 2.17	22.97 ± 1.45	21.86 ± 1.12	21.5 ± 2.13
Prothrombin time, sec	13.2 ± 0.7	15.4 ± 1.1	20.6 ± 5.12	17.2 ± 3.14	14.6 ± 1.45	14.7 ± 1.12
International normalized ratio	1.22 ± 0.08	2.3 ± 1.34	3.14 ± 0.89	1.46 ± 0.52	1.23 ± 0.37	1.24 ± 0.18
Partial thromboplastin time, sec	32.35 ± 5.12	47.44 ± 4.23	57.21 ± 5.18	50.3 ± 4.65	44.92 ± 3.36	39.55 ± 4.63
Fibrinogen, mg/dL	410.1 ± 52.2	407.5 ± 45.3	387.1 ± 38.9	313.7 ± 45.2	279.7 ± 36.7	222.7 ± 39.8
FHB, g/L	0.039 ± 0.021	0.055 ± 0.014	0.093 ± 0.035	0.066 ± 0.024	0.065 ± 0.018	0.068 ± 0.026
Biochemical data (mean ± SD)						
SGOT (ALT), U/L	16.3 ± 2.1	40.9 ± 5.2	28.5 ± 3.3	17 ± 2.9	14 ± 1.8	15.7 ± 2.3
Total bilirubin, Umol/L	2.1 ± 0.3	2.4 ± 0.7	3.9 ± 1.1	2.7 ± 0.8	2.4 ± 0.6	2.1 ± 0.8
Albumin, g/dL	2.6 ± 0.2	2.1 ± 0.9	2.2 ± 0.4	2.0 ± 0.5	2.3 ± 0.6	2.4 ± 0.3
AST, U/L	124.8 ± 89.6	259.4 ± 91.2	170.8 ± 75.6	106.1 ± 57.6	103.5 ± 45.9	102.3 ± 46.8
Glutamyl endopeptidase, U/L	81.3 ± 35.7	65.7 ± 33.6	54.3 ± 22.5	60.7 ± 17.9	64.4 ± 22.1	61.1 ± 24.5
Serum urea nitrogen, mmol/L	5.42 ± 1.32	10.64 ± 2.89	7.36 ± 1.25	6.41 ± 1.10	5.72 ± 0.89	5.33 ± 0.75
Creatinine, Umol/L	76.6 ± 9.32	67.4 ± 10.23	69.1 ± 11.32	70.6 ± 12.69	73.2 ± 11.85	66.6 ± 13.10
Glucose, Umol/L	3.16 ± 0.28	3.65 ± 0.54	3.80 ± 0.32	3.45 ± 0.49	2.54 ± 0.36	3.59 ± 0.41

SGOT (ALT): serum glutamic-oxaloacetic transaminase (alanine aminotransferase); AST: aspartate aminotransferase.
